# In-depth phylogenetic analysis of hepatitis C virus subtype 1a and occurrence of 80K and associated polymorphisms in the NS3 protease

**DOI:** 10.1038/srep31780

**Published:** 2016-08-17

**Authors:** André F. Santos, Gonzalo Bello, Luãnna L. Vidal, Suiane L. Souza, Daiana Mir, Marcelo A. Soares

**Affiliations:** 1 Departamento de Genética, Universidade Federal do Rio de Janeiro, Rio de Janeiro, Brazil; 2 Laboratório de AIDS & Imunologia Molecular, Instituto Oswaldo Cruz, FIOCRUZ, Rio de Janeiro, Brazil; 3 Programa de Oncovirologia, Instituto Nacional de Câncer, Rio de Janeiro, Brazil

## Abstract

HCV genetic diversity is high and impacts disease progression, treatment and drug resistance. HCV subtype 1a is divided in two clades (I and II), and the 80 K natural polymorphism in the viral NS3 protease is prevalent in clade I. Paradoxically, countries dominated by this clade have contrasting frequencies of 80 K. Over 2,000 HCV 1a NS3 sequences were retrieved from public databases representing Europe, Oceania and the Americas. Sequences were aligned with HCV reference sequences and subjected to phylogenetic analysis to investigate the relative presence of different subtype 1a clades and NS3 protease mutations. HCV-1a sequences split into clades I and II. Clade I was further structured into three subclades, IA to C. Sub-clade IA prevailed in the U.S., while subclade IC was major in Brazil. The NS3 80 K polymorphism was associated with subclade IA, but nearly absent in subclades IB and IC, a pattern similarly seen for the 91S/T compensatory mutation. Three HCV-1a-I sub-clades have been identified, with different frequencies in distinct regions. The 80 K and 91A/S mutations were associated with subclade IA, which provide an explanation for the disparities seen in simeprevir resistance profiles of countries dominated by HCV 1a-I, like the U.S. and Brazil.

Since its discovery in 1989 as the causative agent of non-A non-B hepatitis, the hepatitis C virus (HCV), a member of Flaviviridae family, accounts for the infection of approximately 1.6% of the human population worldwide^1^. This virus is characterized by a great genetic diversity that permits its classification into seven genetically distinct genotypes – gen - (1 through 7) and 67 subtypes (a-x)^2^. In adults, HCV gen1 represents almost half (46%) of the total infections, followed by gen3 (22%), 2 and 4 (13% each)^1^.

HCV genetic variability is known to impact disease progression, cancer development, treatment and acquisition of drug resistance. Patients infected with HCV gen1 and 4 have the lowest rates of sustained virological response (SVR) to traditional pegylated interferon and ribavirin when compared to gen2, 3, 5 and 6^3^,^4^, and to overcome that limitation a new generation of direct-acting antivirals like the NS3 protease inhibitors have been developed^5^. Within HCV gen1, subtype 1b has a higher genetic barrier to develop NS3 protease inhibitor (PI) resistance than subtype 1a and, consequently, responds better to PI-based therapy^6^. In 2013, the new PI simeprevir was approved for treating HCV gen 1 and 4 infections. Again, the success therapeutic response of subtype 1a was lower than that of subtype 1b, a phenomenon attributed to the presence of the NS3 polymorphism 80 K in the former^7^.

Recent evidence suggests that HCV subtype 1a can be classified into two distinct genetic clades (I and II) with a non-homogenous geographic distribution^8^,^9^,^10^. Both clades seem to co-circulate at approximately similar prevalence in European countries, while clade I accounts for nearly 75% of the circulating HCV 1a strains in the United States (US)^11^ and >95% of the strains from Brazil^9^,^12^. A disparate prevalence of the NS3 polymorphism 80 K was also observed across different HCV subtype 1a clades. The 80 K polymorphism is present in around 50% of clade I isolates, but is very low prevalent (<3%) in clade II^9^. This may explain the disproportionate occurrence of 80 K in the US when compared to Europe and to other regions of the world^11^. In fact, the 80 K polymorphism has been recently suggested to have arisen in the US^13^.

The association between the HCV subtype 1a clade I and 80 K seems, however, to be more complex than originally envisaged. Despite HCV 1a strains from Brazil are mostly classified within clade I, a very low prevalence of 80 K polymorphism (<7%) has been described in the country^9^,^14^. These apparently conflicting results prompted us to investigate in further detail the amino acid composition of HCV subtype 1a clades worldwide. In the present work, we investigated the phylogenetic relationships of worldwide HCV subtype 1a NS3 sequences, and assessed their association with the 80 K polymorphism, as well as other underlying amino acid substitutions that have been reported as related to the former^13^.

## Results

**Figure 1:**
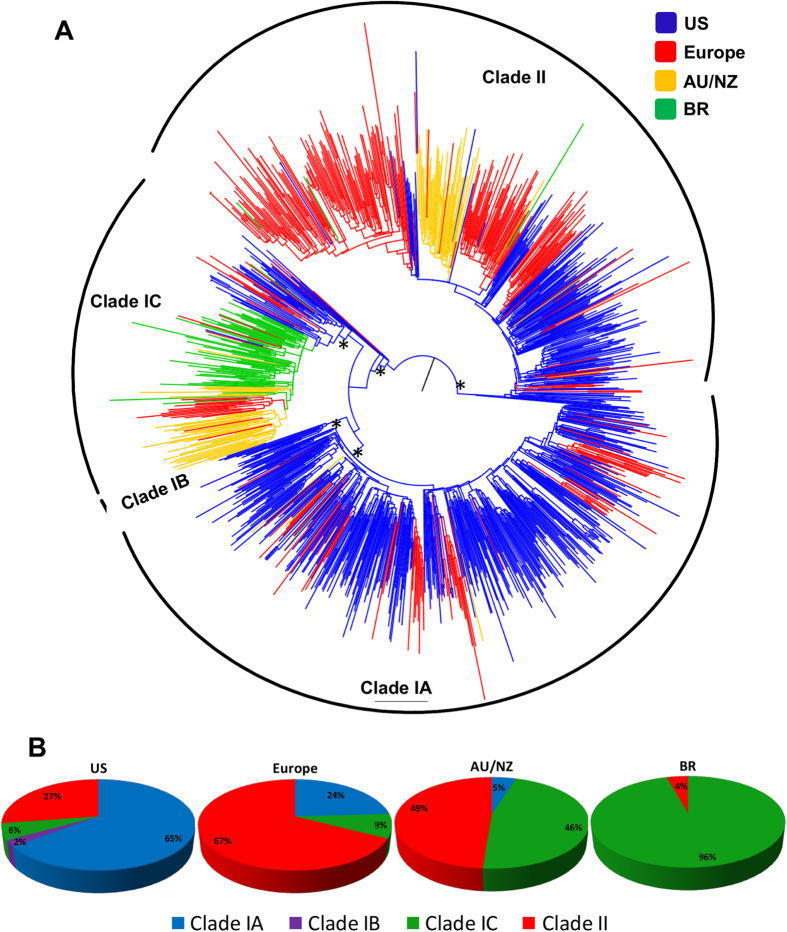
(**A**) Maximum likelihood phylogeny of HCV subtype 1a NS3 gene sequences isolated worldwide showing the geographic distribution of HCV 1a clades and sub-clades. Branches are colored according to the geographic origin of sequences as indicated in the legend (upper right). Arcs indicate the positions of HCV 1a clades and sub-clades. Asterisks point to key nodes with high support (aLRT ≥ 0.90). The tree was rooted at the midpoint. (**B**) Charts depicting the frequency of HCV 1a clades and sub-clades in the United States of America (US), Europe, Australia/New Zeland (AU/NZ) and Brazil (BR).

The nucleotide sequences of NS3 protease from worldwide HCV subtype 1a strains split into clades I and II, as expected (Fig. 1A). Interestingly, we found that clade I was further structured into three well-supported sub-clades that were named clades IA, IB and IC. Sequences from the US branched basal to both clades I and II, as well to sub-clades IA, IB and IC (Fig. 1A). Basal to clade I, a few US and European sequences did not form a specific sub-clade, but are clearly suggestive of being ancestral to the three sub-clades currently observed.

The regional distribution of HCV subtype 1a clades was widely heterogeneous with a clear predominance of clade II in Europe (67%), clade I in the US (73%) and in Brazil (96%), and a comparable frequency of both clades in Oceania (Australia / New Zealand) (Fig. 1B). With respect to clade I sub-clades, the geographic discrepancy was even higher, with clade IA being prevalent in the US (65%) and clade IC in Brazil (96%). Interestingly, clade IB was only found in the US, the only country were all three clade I sub-clades coexist.

The newly found HCV 1a clade I sub-clades also displayed disparate occurrence of natural NS3 polymorphisms related to simeprevir resistance. The proportion of the major resistance mutation 80 K was 64% in clade IA, 0% in clade IB and 3% in clade IC (Table 1). In addition, the presence of this mutation in clade II was uncommon (<1%). The compensatory mutation 91S/T shared similar proportions: the highest in sub-clade IA (59%) and lower in the remaining clade I sub-clades and in clade II (0–8%) (Table 1). However, the compensatory mutation 174N was more prevalent in all three clade I sub-clades I (75–92%) than in clade II (14%) (Table 1).

**Figure 2:**
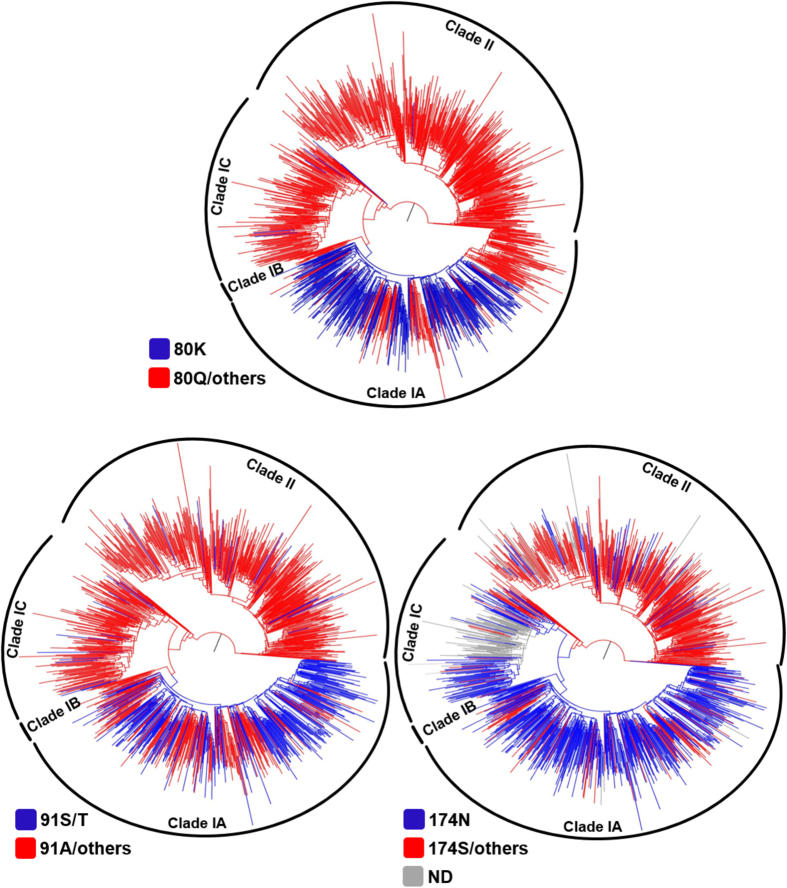
Maximum likelihood phylogenies of HCV subtype 1a NS3 gene sequences with branches colored according to the amino acid signature at positions 80, 91 and 174 of the NS3 protein as indicated in the legend (lower left of each tree). Arcs indicate the positions of HCV 1a clades and sub-clades. The trees were rooted at the midpoint.

When analyzing the appearance of 80 K in the clade I phylogeny, we observed that some basal sequences to this clade harbored that mutation, as well as its occasional emergence in sub-clade IC (Fig. 2). The emergence of 80 K was correlated with basal sequences of sub-clade IA, with some reversions to Q across the phylogeny. The same pattern was observed for the emergence of 91S/T (Fig. 2). The 174N mutation appeared early in all three clade I sub-clades, with some rare amino acid changes to serine or others, while it was rarely found in clade II sequences (Fig. 2).

## Discussion

The subdivision of subtype 1a into two distinct genetic clades has been previously shown^8^. In the present study, we show for the first time the subdivision of clade 1a-I into three well-structured sub-clades (A, B and C). While previous studies have used similar phylogenetic approaches to study the relationships between HCV 1a sequences 8,9,10, they have failed to evidence a substructure within clade subtype 1a clade I, likely due to a limited number^10^ or representativeness^8^,^9^ of sequences from worldwide locations. That was also likely the reason by which a more recent study^11^ has not pinpointed such structure. A previous study with Brazilian HCV NS5A sequences from treatment-naïve subjects evidenced a monophyletic cluster within subtype 1a clade I^15^. Upon the analyses conducted herein, we infer those sequences as belonging to subtype 1a sub-clade IC, corresponding to 96% of all Brazilian subjects infected with subtype 1a from whom sequence information is available.

The majority of HCV subtype 1a sequences from the US and Brazil fell within clade I, while sequences from Oceania belonged to both clades I and II at roughly similar proportion, as described previously^11^,^12^. Clade II represented 67% of European subtype 1a sequences while clade I enclosed 27% of US sequences, frequencies also similar to those previously reported^11^. Some sequences from US and Europe were basal even to the split of the three observed clade I sub-clades, representing the likely ancestors to the whole clade I and corroborating the hypothesis postulated by De Luca *et al.*^11^. However, in our study, US and European sequences were also placed basal to clade II, indicating again a mixed origin of clade II, in contrast to the previous work that showed an unique European origin for that clade^11^.

Several studies suggested previously that the HCV NS3 polymorphism 80 K, which confers resistance to the NS3 inhibitor simeprevir^16^, is heterogeneously distributed within HCV genotype 1, present in 1–43% of HCV subtype 1a and 0–6% in subtype 1b depending of the geographic region analyzed^14^,^17^,^18^. It was been recently proposed that 80 K emerged in the US around 1940–1955 and that sequences harboring that polymorphism grouped together in a monophyletic clade^13^. Herein we classified most of HCV 1a sequences harboring the 80 K mutation within clade IA, while in other clades its prevalence is very low (3% in sub-clade IC, 0% in sub-clade IB and <1% in clade II). In our analyses, 64% of sub-clade IA sequences harbor 80 K, including some sequences branching at the root of that sub-clade. Therefore, our data revealed that the 80 K mutation emerged multiple times during the radiation of HCV subtype 1a clades and sub-clades, including one correlated with the genesis of sub-clade IA. The 80 K mutation was probably fixed at the base of sub-clade IA with further genetic reversion to 80Q in a number of viral isolates.

The NS3 A91S/T and S174N polymorphisms were strongly associated with 80 K^13^. In our study, A91S/T appeared in a similar proportion to that of 80 K in sub-clade IA (59% and 64%, respectively), while it was rare in other clade I sub-clades and in clade II (0–8%). The co-occurrence of A91S/T and 80 K did not differ from a random association (data not shown). On the other hand, 174N was found in high proportion in all clade I sub-clades (75–92%), but less frequently in clade II (14%). In this case, an association between 174N and 80 K would be obvious in clade IA, where the majority of the sequences harbor both polymorphisms, but not in clades IB and IC, with high frequency of 174N but no 80 K. Our data suggest that these mutations are not necessarily biologically associated with 80 K, but further association studies are necessary to establish the co-evolutionary patterns between these two polymorphisms and 80 K in HCV NS3.

Patients infected with HCV subtype 1a carrying 80 K present a reduced sustained virological response compared to those not carrying the polymorphism when treated with simeprevir-containing regimens^7^. Because of that, American and European guidelines for treatment of HCV infection currently indicate genotyping of patients infected with subtype 1a for 80 K detection prior to simeprevir usage^19^,^20^. Recently, 80 K was shown to correlate with HCV subtype 1a clade I strains^11^. Now we show that the majority of 80 K-harboring strains are classified into sub-clade IA, an observation that is congruent with the heterogeneity in the prevalence of 80 K in different parts of the world (1.3% in Brazil; 43% in the US and approximately 14% in Europe)^14^. Despite all these areas are dominated by clade I strains, the prevalence of sub-clade IA is null in Brazil, while it accounts for 65% and 24% in the US and Europe, respectively.

In conclusion, we showed that HCV subtype 1a clade I is further structured into three definite sub-clades (IA, IB and IC) with sequences geographically segregated, suggesting a founder effect in some cases, like sub-clade IC in Brazil and Oceania. We also show that the presence of the NS3 80 K variation is biased in specific sub-clades, being found majorly in sub-clade IA sequences. These data may improve induced treatment access with simeprevir in areas where subtype 1a sub-clades IB and IC prevail without the need for previous virus genotyping assessment.

## Methods

### Sequences

A total of 2,185 NS3 protease sequences from treatment-naïve patients infected with HCV subtype 1a of different countries has been described previously^14^ and used in the present study. To obtain reliable phylogenetic relationships among sequences, only those with the complete HCV NS3 protease sequence were selected and used in the herein analyses, totaling 1,140 sequences: 363 from Europe, 573 from North America (US), 110 from South America (Brazil) and 94 from Oceania. Sequences from Africa and Asia were limited in number and were excluded from the analyses. Sequence alignment was performed with ClustalX v.2.0 and the BioEdit platform v.7.0.5.3 was used for editing and sequence analysis^21^. References sequences of HCV subtype 1a of clades I and II were extracted from Pickett *et al.*^8^.

### Phylogenetic analysis

To investigate the relative prevalence of different HCV subtype 1a clades and protease mutations across different geographic regions we performed a Maximum Likelihood (ML) phylogenetic analysis of HCV 1a NS3 sequences. The ML phylogenetic tree was inferred using PhyML program^22^,^23^, employing the GTR+I+G nucleotide substitution model selected by the Akaike information criterion implemented in jModelTest^24^, the subtree pruning and regrafting (SPR) branch swapping algorithm of tree search, and the approximate likelihood ratio test (aLRT) of statistical support for individual nodes.

## Additional Information

**How to cite this article**: Santos, A. F. *et al.* In-depth phylogenetic analysis of hepatitis C virus subtype 1a and occurrence of 80K and associated polymorphisms in the NS3 protease. *Sci. Rep.* **6**, 31780; doi: 10.1038/srep31780 (2016).

## Figures and Tables

**Table 1:** Frequency of different amino acid signatures at positions 80, 91 and 174 of the NS3 protein across HCV 1a clades and sub-clades.

NS3 position	Polymorphism	Clade IA (%)	Clade IB (%)	Clade IC (%)	Clade II (%)
80	Q	34	92	94	97
	K	64	0	3	0
	Others	2	8	3	3
91	A	40	100	88	94
	S/T	59	0	8	4
	Others	1	0	4	2
174	S	17	8	21	72
	N	82	92	75	14
	Others	1	0	4	14
